# Cellular Localization and Biochemical Characterization of a Chimeric Fluorescent Protein Fusion of *Arabidopsis* Cellulose Synthase-Like A2 Inserted into Golgi Membrane

**DOI:** 10.1155/2014/792420

**Published:** 2014-01-14

**Authors:** Monica De Caroli, Marcello S. Lenucci, Gian-Pietro Di Sansebastiano, Michela Tunno, Anna Montefusco, Giuseppe Dalessandro, Gabriella Piro

**Affiliations:** Dipartimento di Scienze e Tecnologie Biologiche ed Ambientali, Università del Salento (DiSTeBA), Provinciale Lecce-Monteroni, 73100 Lecce, Italy

## Abstract

*Cellulose synthase*-*like* (*Csl*) genes are believed to encode enzymes for the synthesis of cell wall matrix polysaccharides. The subfamily of *CslA* is putatively involved in the biosynthesis of **β**-mannans. Here we report a study on the cellular localization and the enzyme activity of an *Arabidopsis* CslA family member, AtCslA2. We show that the fluorescent protein fusion AtCslA2-GFP, transiently expressed in tobacco leaf protoplasts, is synthesized in the ER and it accumulates in the Golgi stacks. The chimera is inserted in the Golgi membrane and is functional since membrane preparations obtained by transformed protoplasts carry out the *in vitro* synthesis of a ^14^C-mannan starting from GDP-d-[U-^14^C]mannose as substrate. The enzyme specific activity is increased by approximately 38% in the transformed protoplasts with respect to wild-type. Preliminary tests with proteinase K, biochemical data, and TM domain predictions suggest that the catalytic site of AtCslA2 faces the Golgi lumen.

## 1. Introduction

Cellulose, hemicelluloses, and pectins are the main structural polysaccharides of the plant cell wall. Glycosyltransferases (Gts) are the enzymes involved in the synthesis of these polysaccharides and other glycans. Plasma membrane rosette complexes are the Gts involved in the synthesis of cellulose [1] whereas Gts located in the Golgi apparatus are responsible for the pectin and hemicellulose synthesis [2–4]. *Cellulose synthase* (*CesA*) genes were first identified in cotton fibers [5]. Later, in addition to *CesA* genes, *Arabidopsis* genome sequencing revealed a large gene family with homology to *CesA* [6] named *cellulose synthase-like* (*Csl*).

Two classes of Gts are mainly involved in cell wall polysaccharide biosynthesis: the multimembrane spanning and the type II transmembrane (TM) Gts, the latter consisting of a short N-terminal domain on the cytoplasmic side of the membrane, a single TM domain, a stem of variable length, and a large globular C-region containing the catalytic domain. Multi-membrane spanning Gts (cellulose and callose synthases) are typically associated with the plasma membrane, while both Gts classes type II and multi-membrane spanning have been found in Golgi stacks where they are likely involved in the biosynthesis of complex *β*-glycan polymers [7–9]. The Golgi multi-membrane spanned Csl proteins have several TM domains close to the N-terminus followed by a large hydrophilic loop containing the predicted active site motif D,D,D,QxxRW, highly conserved in CesA and Csl family members; a second hydrophilic loop and a variable number of TM domains, beside the C-terminus, are also present [6, 10, 11]. Depending on the number of N-terminus TM helices, the catalytic domain may face the cytoplasmic (even) or the luminal (odd) side of Golgi membranes [7, 12]. The first type, like CesA, uses substrate donor NDP-sugars of the cytosolic pool; the second type uses NDP-sugars transported into the Golgi by specific carrier proteins [13, 14].

Until now, little evidence for the functional role of *Csl* genes has been collected. Among the six groups of the *Csl* gene family identified, only few members of *CslA* and *CslC* have been functionally characterized suggesting their respective involvement in (gluco)mannan [15, 16] and xyloglucan synthesis [17]. A role in the synthesis of mixed *β*-glucan has been proposed for members of *CslG* and *CslF* groups [18, 19].

In this study we constructed a fluorescent fusion of an *Arabidopsis* CslA family member, AtCslA2-GFP, to identify its final localization in a heterologous system represented by tobacco leaf protoplasts. The chimera was localized in the Golgi stacks in a functional organization since an increase in mannan synthesis was quantified in transformed protoplasts. Combining confocal observation, biochemical data, and TM domain predictions, the topologic organisation of the chimera in the Golgi membrane is discussed.

## 2. Material and Methods

### 2.1. Plasmid Construction

The cDNA coding for AtCslA2 was purchased from The *Arabidopsis* Information Resource (TAIR, Ohio State University). The cDNA sequence was amplified with primers Man01 (5′-ggagcccggatccatggacggtg-3′) and Man02 (5′-caactgctagctcgggacataagtccc-3′) to introduce, respectively, *Bam*HI and *Nhe*I restriction sites. The *Bam*HI/*Nhe*I fragment was introduced into a GFP-containing vector PGIP2-GFP [20] to construct AtCslA2-GFP. The construct was confirmed by sequencing (PRIMM srl, Milan, Italy).

### 2.2. Protoplast Preparation, Transformation, and Drug Treatments

Tobacco protoplasts were prepared from the leaves of *Nicotiana tabacum* cv SR1 plants and transiently transformed as described by [21]. Equivalent quantities (20 *μ*g) of plasmids were used for the colocalization experiments. The fluorescent pattern of over 1200 cells was analysed. Brefeldin A (10 *μ*g/mL; Sigma-Aldrich) or cycloheximide (30 *μ*g/mL; Sigma-Aldrich) was added into the incubation medium for 2 h before confocal analyses.

### 2.3. Laser Scanning Confocal Microscopy

Protoplasts transiently expressing fluorescent constructs were observed by a laser scanning confocal microscope (LSM 710 Zeiss) in their culture medium at different times after transformation. To detect GFP fluorescence, a 488 nm argon ion laser line was used, and the emission was recorded with 505–530 nm filter set; RFP was detected with a 560–615 nm filter set after He–Ne laser excitation at 543 nm, while chlorophyll epifluorescence was detected with the filter set for tetramethylrhodamine isothiocyanate (TRITC; >650 nm) and eliminated. The power of each laser line, the gain, and the offset were identical for each experiment so that the images were comparable. Appropriate controls were performed to exclude the possibility of crosstalk between the two fluorochromes before image acquisition.

### 2.4. Protein Extraction, Immunoblotting Analyses, Cell Lysis, and Phase Separation

Extracellular and intracellular protein fraction, cell lysis, phase separation, and immunoblot analyses were performed as previously described [20]. Protoplasts were harvested by gentle centrifugation at 65 ×g without break, after dilution of the incubation medium with two volumes of W5. The medium was concentrated by filtration on Centricon Plus 20 (Amicon) to obtain the extracellular protein fraction (OUT). The protoplasts were suspended in homogenization buffer (40 mM Hepes-NaOH buffer pH 7.5, 10 mM imidazole, 1 mM benzamidine, 5 mM 6-amino-hexanoic acid, 10 mM dithiothreitol, and 1 mM phenylmethylsulfonyl fluoride) and sonicated. The homogenate was centrifuged at 800 ×g for 10 min at 4°C to remove cell wall debris and the supernatant was precipitated with 80% acetone at −20°C (three times) to obtain the protein intracellular fraction (IN) that includes endomembrane compartments and organelles. Protein concentration was determined according to [22]. Identical amounts of total protein from each subcellular preparation were subjected to SDS-PAGE electrophoresis [23] and immunoblotting analyses. After electrophoresis, protein samples were transferred to a nitrocellulose membrane (Hybond-C extra: GE Healthcare) and blocked with 5% skim-milk powder in TBS (20 mM Tris-HCl pH 7.5; 500 mM NaCl; 5% w/v milk powder) for 2 h. Anti-GFP (1 : 5000 v/v) (Molecular Probes) was used in TBS + 1% skimmed-milk powder. Each protein fraction was visualized after ECL (ECL western blotting analysis system, GE Healthcare) chemodetection. Cell lysis and phase separation were carried out as described by [24] modified by [20]. To test AtCslA2 solubility in Triton X-114, AtCslA2-GFP transformed protoplasts were freeze-thawed twice in 10 mM Tris-buffer pH 7.5 containing 0.15 M NaCl and 1 mM EDTA and centrifuged at 800 ×g for 10 min. The 800 ×g supernatant was adjusted to 1.5 mL with Tris-buffer (pH 7.5) and Triton X-114 was added into a final concentration of 1% v/v.

### 2.5. Enzyme Assay

To assay mannan synthase activity, protoplasts were suspended and sonicated in 0.1 M sodium-phosphate buffer (pH 7.2) containing 10 mM MgCl_2_, 1 mM dithiothreitol, 0.4 M sucrose, and 1% bovine serum albumin. The resulting homogenate was centrifuged at 800 ×g for 10 min at 2°C. The 800 ×g supernatant was further centrifuged at 100000 ×g for 60 min at 2°C to obtain a pellet consisting of membranous material (total membrane fraction). This pellet was suspended in 0.5 mL of 0.1 M sodium-phosphate buffer (pH 7.2) as described above, homogenized, and immediately stored in Eppendorf tubes under liquid nitrogen until use. Mannan synthase assays were performed as described by [25]. Standard reaction was carried out in a final volume of 50 *μ*L containing 0.20 nmol of GDP-d-[U-^14^C]mannose (approximately 1500 Bq), 20 nmol of GDP-d-mannose, and 10 *μ*L of total membrane fraction in 0.1 M sodium-phosphate buffer (pH 7.2). Reactions were performed at 27°C for 30 min and stopped by adding 500 *μ*L of 70% ethanol. Ethanol insoluble molecules, including ^14^C-polymer, were precipitated by centrifugation at 10000 ×g for 5 min. The pellet was rewashed five times with 70% ethanol in order to remove unreacted radiolabelled precursors. To verify the presence of a 2-epimerase activity, the incubation medium, depleted of polymers, was paper-electrophoresed (pH 3.5) [26] and the eluted GDP-sugars were hydrolysed in 0.1 M HCl and paper-chromatographed (solvent A). When the effect of proteinase K and Triton X-100 was tested, equal aliquots of total membrane fraction were incubated with 400 *μ*g/mL Proteinase K with or without the addition of 1% Triton X-100 for 30 min at 25°C. The reactions were stopped by the addition of 500 *μ*L of 200 mM PMSF.

### 2.6. Acid and Enzymic Hydrolysis

Total acid hydrolysis of the ^14^C-polymer was carried out in 3% (w/w) H_2_SO_4_ at 120°C, 103 kPa, for 1 h. The hydrolysates were neutralised by a bicarbonate form of amberlite IR-45 (OH) resin, rotary-evaporated to dryness under reduced pressure, and dissolved in 30 *μ*L of H_2_O and an aliquot used for paper chromatography. Enzymic hydrolysis with *β*-mannanase from *Bacillus subtilis* or *Penicillium wortmannin* was performed as described by [27].

### 2.7. Paper Chromatography, Paper Electrophoresis, and Radioactive Counting Procedure

Descending paper chromatography was performed on Whatman no. 1 paper and developed with Solvent A: ethyl acetate-pyridine-water (8 : 2 : 1, by vol.). Paper electrophoresis was carried out on Whatman no. 1 paper in acetic acid (8%, v/v) and formic acid (2%, v/v) buffer, pH 2.0, 5 kV for 45 min to separate GDP-sugars and sugar phosphates from polysaccharides and neutral material [21]. To separate NDP-sugars, paper electrophoresis was carried out in pyridine 5% (v/v) acetic acid buffer pH 3.5, 5 kV for 30–45 min [26]. Detection of the markers and radioactive counting procedure were performed as described by [25].

### 2.8. Statistical Analyses

The biochemical data from wild-type and AtCslA2-GFP transformed tobacco leaf protoplasts were compared using Student's *t*-test and values were expressed as means ± standard deviation of at least three independent replicated experiments (*n* = 3). SigmaStat version 3.11 software (Systat Software Inc.) was used for statistical analysis. *P* value of less than 0.05 was considered to indicate statistical significance.

## 3. Results

### 3.1. Cellular Localization of AtCslA2 Expressed in Tobacco Leaf Protoplasts

**Figure 1:**
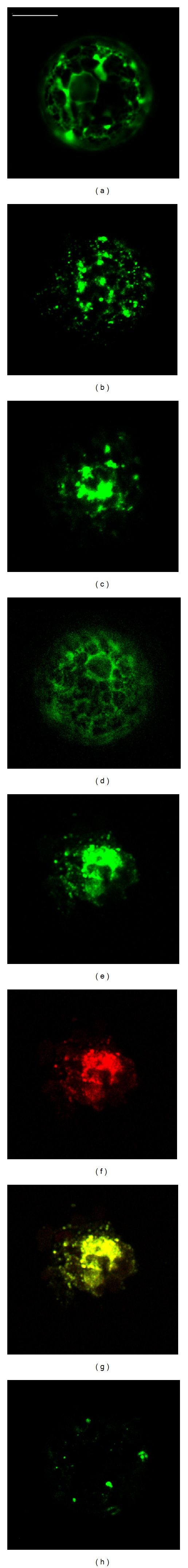
Subcellular localization of AtCslA2-GFP. AtCslA2-GFP transiently expressed in tobacco leaf protoplasts labels ER and Golgi stacks (a). Brefeldin A determines the aggregation of the Golgi and the redistribution of the fluorescence in the ER (b–d). AtCslA2-GFP colocalises with the Golgi marker ST52-mRFP (e–g). Golgi stacks are the only fluorescent compartment labelled after 2 h of cycloheximide treatment (h). Scale bar: 20 *μ*m.

A C-terminal fusion of the full-length coding region of *AtCslA2* to the green fluorescent protein (GFP) was constructed and transiently expressed in tobacco leaf protoplasts to investigate its subcellular localization. Laser confocal scanning microscopy revealed fluorescence in a cortical reticulate network and some high mobile punctate structures (Figure 1(a)), suggesting the chimera localization in the ER and Golgi compartments.

Treatment with Brefeldin A, a lipophilic fungal toxin known to induce disassembly of the Golgi stacks among other effects [28], determined the aggregation of the punctate structures (Figures 1(b) and 1(c)) and, during time, the redistribution of the fluorescence into the tubular and cortical network (Figure 1(d)). This suggested that AtCslA2-GFP may be localized in the Golgi stacks. To further demonstrate this localization, the chimera was coexpressed with ST52-mRFP, a *trans*-Golgi stack marker [20, 29]. A complete colocalization of the green and red fluorescence was observed (Figures 1(e)–1(g)), evidencing that both ST52-mRFP and AtCslA2 accumulated in the Golgi stacks. The final localization of the chimera in the Golgi stacks was also confirmed by treating AtCslA2-GFP transiently transformed protoplasts with the protein synthesis inhibitor cycloheximide. After 2 h of treatment, the punctate structures were the only fluorescent compartments evidenced (Figure 1(h)). Altogether these data demonstrate that GFP-tagged AtCslA2 protein is cotranslationally inserted into ER to further reach the Golgi apparatus, which represents its stable localization.

### 3.2. AtCslA2-GFP Is Integrated in the Golgi Membranes

**Figure 2:**
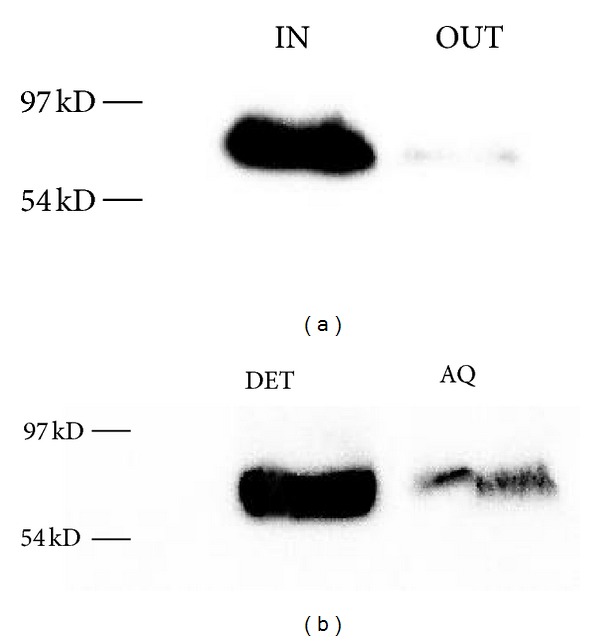
AtCslA2-GFP is insoluble in the detergent Triton X-114. Western blot analysis of protein fractions (intracellular, IN; incubation medium, OUT) obtained from AtCslA2-GFP transformed protoplasts (a). AtCslA2-GFP is Triton X-114 insoluble and is mainly recovered in the detergent phase (DET) a small contamination of the chimera was present in the aqueous phase (Aq) (b). Bands were detected using anti-GFP serum.

The presence of the chimera in the intracellular protein fraction, including the endomembrane system, was confirmed by immunoblot analyses of protein extracted from the transformed protoplasts using an anti-GFP antibody (Figure 2(a)). The protein band shows the expected molecular mass of 88.5 kDa. A very small amount of the chimera was detected in the protein fraction of the medium. The amino acid (aa) sequence analysis of the *AtCslA2* cDNA product predicted a polypeptide of 534 aa showing four TM domains and two hydrophilic regions, between the first and second and the third and fourth TM domains (TMHMM server v. 2.0., http://www.cbs.dtu.dk/services/TMHMM/). To ascertain the insertion of the chimera into the lipid bilayer, its solubility in the nonionic detergent Triton X-114 was analysed. After extraction and phase separation of the total AtCslA2 transformed protoplast proteins, the major percentage of the chimera was detected in the detergent phase confirming that it was an integral membrane protein (Figure 2(b)).

### 3.3. Biochemical Characterization of AtCslA2-GFP Expressed in Tobacco Leaf Protoplasts

*AtCslA2* is a member of *Arabidopsis CslA* gene family encoding for a putative mannan synthase. To determine whether AtCslA2-GFP was functional in tobacco protoplasts, we tested its enzymatic activity isolating the total membrane fraction from the homogenates of wild-type and AtCslA2-GFP transiently transformed protoplasts and incubating them in the presence of GDP-d-[U-^14^C]mannose as substrate. After 30 min, the radioactivity incorporated in the 70% ethanol precipitated product was measured and the enzymatic activity associated with the membranes was calculated (Table 1). Radioactive polymers were synthesized either from wild-type or transformed protoplasts. ^14^C-polymers were immobile when electrophoresed in acetic acid/formic acid buffer (pH 2.0) and were not eluted from Whatman no. 1 paper after several washing either with H_2_O or with mixtures of chloroform-methanol (1 : 1, v/v) and chloroform-methanol-water (3 : 48 : 47; 10 : 10 : 3, v/v). Total acid hydrolysis and *β*-mannanase treatment of the ^14^C-polymers released only mannose as radioactive sugar (paper chromatography, solvent system A). No other radioactive sugars were detected when the incubation medium was analysed for the presence of a GDP-mannose 2-epimerase activity (data not shown). These results demonstrated the presence of a *β*-mannosyl transferase activity in tobacco leaf protoplasts and an appreciable, highly statistically significant (*P* = 0.004) increase of this activity, approximately 38%, in AtCslA2-GFP transformed protoplasts indicating that the inserted chimera functions properly in the Golgi membranes.

**Figure 3:**
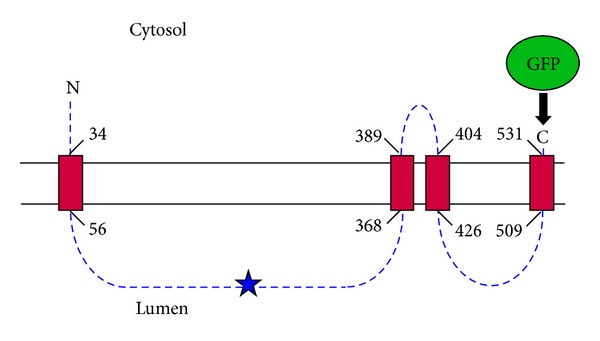
Hypothetical model of membrane topology of AtCslA2-GFP. Four transmembrane domains, predicted by TMHMM server, are reported in red, numbers mark location of the amino acids. Two hydrophilic region are predicted, the larger of which contains the predicted active site (blue star) and faces the Golgi lumen.

Based on TM domain prediction (TMHMM server), AtCslA2 active site should face the Golgi lumen. To ascertain this hypothesis, mannan synthase activity was tested in the presence of proteinase K, with or without Triton X-100. The enzymatic activity was drastically reduced (*P* < 0.001 with respect to untreated AtCslA2-GFP transformed protoplasts) when membranes were permeabilized with detergent during proteinase K treatment (Table 1). The results indicate that the catalytic site of AtCslA2 faces the lumen of the Golgi stacks, according to bioinformatic predictions. A hypothetical model of the polypeptide topology based on the locations of the predicted TM domains is reported in Figure 3.

## 4. Discussion

In order to define the intracellular localization of AtCslA2 (putative mannan synthase encoded by a member of *Arabidopsis CslA* gene family), a fusion with GFP was constructed (AtCslA2-GFP) and transiently expressed in tobacco leaf protoplasts. The fluorescent chimera obtained was retained in the intracellular compartments labelling ER and Golgi stacks. The presence of the chimera in the ER was due to its synthesis and transit in this compartment. In fact, Golgi stacks were the stable and final localization of the chimera, as evidenced by a notable colocalization with the Golgi marker ST52-mRFP (Figures 1(e)–1(g)) and the disappearance of fluorescence from ER compartment in the presence of the protein inhibitor cycloheximide (Figure 1(h)). Furthermore, as previously described for other Golgi resident proteins, Brefeldin A induced a redistribution of the chimera from Golgi stacks to the ER (Figures 1(b)–1(d)) [29].

AtCslA2 is predicted to have four transmembrane (TM) domains (TMHMM server) and therefore it should be an integral membrane protein; we validated this prediction demonstrating its insolubility in the non-ionic detergent Triton X-114. Bioinformatic tools predict the first TM domain as very close to the N-terminus (see scheme in Figure 3) followed by a large hydrophilic region; a second hydrophilic region separates the third from the fourth TM domain. The last TM domain is very close to the C-terminus where GFP was fused. AtCslA2 seems to have a membrane topology similar to that of CesA, callose synttase (CalS), and the other members of Csl family. All of them contain multiple TM domains clustered in two regions with a large hydrophilic central loop containing the putative catalytic site [1, 30]. The catalytic domain faces the cytoplasm in CesA and CalS families [7, 30] and may be located in either the cytosol or the Golgi lumen in Csl proteins [31]. The *Arabidopsis* CslD2 [32] and CslC4 [33] are examples of Csl proteins with a catalytic domain facing the cytoplasmic side of the Golgi showing a topologic organization similar to CesA. On the contrary, *β*-mannan synthases of guar seeds (CtManS) [15] and *Arabidopsis* (CslA9) [33] are predicted to have the catalytic domain within Golgi cisternae. The number and location of the predicted TM domains (1 TM helice close to the N-terminus) in AtCslA2 suggest that the large hydrophilic loop containing the putative catalytic domains likely faces the Golgi lumen. A second hydrophilic region between the third and fourth TM domain is predicted for AtCslA2 as well as for CtManS [15]. This domain is reported to be much longer than any corresponding inter-TM domains toward the C-terminus in CesA [34] and is predicted to be in the Golgi lumen, on the same side of the catalytic domain for both CtManS and AtCslA2. A role in interacting with a galactosyl transferase, that adds galactose onto a mannan backbone, has been hypothised for this domain in CtManS [15].

Characterization and assignment of the specific transglycosylase activity to a glycosyltransferase have always been difficult when either a biochemical or a genetic approach was applied to the problem. Heterologous expression has provided a valid method to determine the enzymatic activity of plant Gts. Heterologous expression in *Drosophila* Schneider 2 (S2) cells of a group of *Csl* genes from *Arabidopsis*, rice, loblolly pine, and *Physcomitrella patens* has allowed to determine a *β*-mannan synthase activity for several members of *CslA* gene family [16, 35]. Also a guar putative *ManS* gene was expressed in embryogenic soybean suspension-culture cells to ascertain its involvement in the synthesis of galactomannans [15]. In the present study we transiently expressed AtCslA2-GFP fusion protein in tobacco leaf protoplasts and analysed its catalytic activity in the total membrane fraction in the presence of GDP-d-[U-^14^C]mannose as substrate. Membrane preparations from untransformed tobacco protoplasts showed an appreciable mannan synthase activity (370 nmol·min/mg of proteins) which increased by approximately 38% in the transformed cells. Although gymnosperms contain higher amounts of (galacto)mannans and/or (galacto)glucomannans with respect to angiosperms, the presence of mannans as structural and storage polysaccharides in plants and algae is well documented [36–39]. A constitutive *β*-mannan synthase activity in tobacco leaf is, therefore, not surprising. The increased *β*-mannan synthase activity in transformed protoplasts clearly indicated a proper folding and a correct function of the chimera into Golgi membranes, despite the presence of GFP as fluorescent tag. A mannan synthase activity has been reported for proteins encoded by *CslA1* and *CslA9* genes from *Arabidopsis* expressed in *Drosophila* S2 cells with an enzymatic activity valuable in the order of pmole/mg of protein [35]. The heterologous system represented by tobacco protoplasts, despite the constitutive background, seems to be more efficient than animal cells since a specific activity in the order of nmol/mg of protein was related to AtCslA2-GFP overexpression.

The chemical characterization of the radiolabelled polymeric material obtained from the *in vitro* incubation of the total membrane fractions isolated from both wild-type and AtCslA2-GFP transformed protoplasts with GDP-d-[U-^14^C]mannose demonstrated that it consisted mainly of polysaccharide whose complete acid hydrolysis gave only mannose as radioactive glycosyl residue. No other sugars were detected either in the polymer or in the reaction mixture indicating that GDP-mannose was not 2-epimerised into GDP-glucose. This result agrees with previously data showing the absence of a 2-epimerase activity, in a membrane system isolated from pea stem, which leads to the synthesis of mannans using GDP-mannose as substrate [25]. On the contrary, an epimerase activity was found in particulate enzymic preparation isolated from pine cambial and xylem cells which leads to the synthesis of glucomannans using only GDP-mannose as substrate [27]. The presence of a GDP-mannose 2-epimerase activity in our system would allow us to easily determine the possible involvement of AtCslA2 in glucomannan biosynthesis by the presence of both d-[U-^14^C]mannose and d-[U-^14^C]glucose residues after complete acid hydrolysis of polysaccharides. We did not test transformed protoplast membrane preparations in the presence of both radioactive GDP-mannose and GDP-glucose and/or UDP-galactose; therefore we cannot exclude that *AtCslA2* gene may have a role in the synthesis of (galacto)mannans or (galacto)glucomannans. A role in (galacto)mannan and (galacto)glucomannan synthesis has been evidenced for several CslA gene products from *Arabidopsis*, guar, and poplar [15, 16, 35, 40–43].

## 5. Conclusions

The present study extends previous analyses on *CslA* gene products demonstrating that AtCslA2 brings about the synthesis of *β*-mannan. The heterologous expression of the fluorescent chimera AtCslA2-GFP in tobacco leaf protoplasts can be considered an efficient experimental system that allows either to follow the correct final localization of the chimera or to test its enzymatic activity. The possibility to overexpress gene products involved in the synthesis of mannan, glucomannan and/or galactoglucomannan, may help to better understand how the encoded proteins operate to synthesize these polysaccharides. This information may have relevant consequences for several technological aspects such as the production of biofuel and gums of high value.

## Figures and Tables

**Table 1:** Mannan synthase activity in wild-type and AtCslA2-GFP transformed tobacco protoplasts and effect of protease K with or without Triton X-100. The reaction mixture contained the following: 0.20 nmol of GDP-D-[U-^14^C]mannose, 20 nmol GDP-D-mannose, and 10 *μ*L (130 *μ*g of protein) of total membrane fraction isolated from wild-type and AtCslA2-GFP transformed tobacco protoplasts, in 0.1 M sodium-phosphate buffer, pH 7.2, in a total volume of 50 *μ*L. Reaction time at 27°C was 30 min. Data are the means ± standard deviation of three independent replicates (*n* = 3).

Protoplasts	Bq	Specific activity (nmol·min/mg of protein)
Wild-type	3.9 ± 0.2	370 ± 25
AtCslA2-GFP transformed	5.4 ± 0.3	510 ± 32
+ Proteinase K	4.6 ± 0.3	438 ± 30
+ Triton X-100	2.2 ± 0.1	209 ± 16
+ Proteinase K + Triton X-100	0.8 ± 0.1	76 ± 5

## References

[1] Doblin MS, Kurek I, Jacob-Wilk D, Delmer DP (2002). Cellulose biosynthesis in plants: from genes to rosettes. *Plant and Cell Physiology*.

[2] Cosgrove DJ (2005). Growth of the plant cell wall. *Nature Reviews Molecular Cell Biology*.

[3] Sandhu APS, Randhawa GS, Dhugga KS (2009). Plant cell wall matrix polysaccharide biosynthesis. *Molecular Plant*.

[4] Liepman AH, Wightman R, Geshi N, Turner SR, Scheller HV (2010). Arabidopsis—a powerful model system for plant cell wall research. *Plant Journal*.

[5] Delmer DP (1999). Cellulose biosynthesis: exciting times for a difficult field of study. *Annual Review of Plant Biology*.

[6] Richmond TA, Somerville CR (2000). The cellulose synthase superfamily. *Plant Physiology*.

[7] Doblin MS, Vegara CE, Read S, Newbigin E, Bacic A, Rose JKC (2003). Plant cell wall biosynthesis: making the bricks. *Annual Plant Reviews, The Plant Cell Wall*.

[8] Scheible W-R, Pauly M (2004). Glycosyltransferases and cell wall biosynthesis: novel players and insights. *Current Opinion in Plant Biology*.

[9] Liepman AH, Cavalier DM (2012). The cellulose synthase like A and cellulose synthase like C families: recent advances and future perspectives. *Frontiers in Plant Science*.

[10] Saxena IM, Brown RM, Fevre M, Geremia RA, Henrissat B (1995). Multidomain architecture of *β*-glycosyl transferases: Implications for mechanism of action. *Journal of Bacteriology*.

[11] Richmond TA, Somerville CR (2001). Integrative approaches to determining Csl function. *Plant Molecular Biology*.

[12] Oikawa A, Lund CH, Sakuragi Y, Scheller HV (2013). Golgi-localized enzyme complexes for plant cell wall biosynthesis. *Trends in Plant Science*.

[13] Norambuena L, Marchant L, Berninsone P, Hirschberg CB, Silva H, Orellana A (2002). Transport of UDP-galactose in plants. Identification and functional characterization of AtUTr1, an Arabidopsis thaliana UDP-galactose/UDP-glucose transporter. *The Journal of Biological Chemistry*.

[14] Reyes F, Orellana A (2008). Golgi transporters: opening the gate to cell wall polysaccharide biosynthesis. *Current Opinion in Plant Biology*.

[15] Dhugga KS, Barreiro R, Whitten B (2004). Guar seed *β*-mannan synthase is a member of the cellulose synthase super gene family. *Science*.

[16] Liepman AH, Wilkerson CG, Keegstra K (2005). Expression of cellulose synthase-like (Csl) genes in insect cells reveals that CslA family members encode mannan synthases. *Proceedings of the National Academy of Sciences of the United States of America*.

[17] Cocuron J-C, Lerouxel O, Drakakaki G (2007). A gene from the cellulose synthase-like C family encodes a *β*-1,4 glucan synthase. *Proceedings of the National Academy of Sciences of the United States of America*.

[18] Burton RA, Wilson SM, Hrmova M (2006). Cellulose synthase-like CslF genes mediate the synthesis of cell wall (1,3;1,4)-*β*-D-glucans. *Science*.

[19] Doblin MS, Pettolino FA, Wilson SM (2009). A barley cellulose synthase-like CSLH gene mediates (1,3;1,4)-*β*-D- glucan synthesis in transgenic Arabidopsis. *Proceedings of the National Academy of Sciences of the United States of America*.

[20] De Caroli M, Lenucci MS, Di Sansebastiano G-P, Dalessandro G, De Lorenzo G, Piro G (2011). Protein trafficking to the cell wall occurs through mechanisms distinguishable from default sorting in tobacco. *Plant Journal*.

[21] Leucci MR, Di Sansebastiano G-P, Gigante M, Dalessandro G, Piro G (2007). Secretion marker proteins and cell-wall polysaccharides move through different secretory pathways. *Planta*.

[22] Bradford MM (1976). A rapid and sensitive method for the quantitation of microgram quantities of protein utilizing the principle of protein dye binding. *Analytical Biochemistry*.

[23] Laemmli UK (1970). Cleavage of structural proteins during the assembly of the head of bacteriophage T4. *Nature*.

[24] Lisanti MP, Caras IW, Gilbert T, Hanzel D, Rodriguez-Boulan E (1990). Vectorial apical delivery and slow endocytosis of a glycolipid-anchored fusion protein in transfected MDCK cells. *Proceedings of the National Academy of Sciences of the United States of America*.

[25] Piro G, Zuppa A, Dalessandro G, Northcote DH (1993). Glucomannan synthesis in pea epicotyls: the mannose and glucose transferases. *Planta*.

[26] Pacoda D, Montefusco A, Piro G, Dalessandro G (2004). Reactive oxygen species and nitric oxide affect cell wall metabolism in tobacco BY-2 cells. *Journal of Plant Physiology*.

[27] Dalessandro G, Piro G, Northcote DH (1988). A membrane-bound enzyme complex synthesising glucan and glucomannan in pine tissues. *Planta*.

[28] Ritzenthaler C, Nebenführ A, Movafeghi A (2002). Reevaluation of the effects of brefeldin a on plant cells using tobacco bright yellow 2 cells expressing golgi-targeted green fluorescent protein and copi antisera. *Plant Cell*.

[29] Saint-Jore-Dupas C, Nebenführ A, Boulaflous A (2006). Plant *N*-glycan processing enzymes employ different targeting mechanisms for their spatial arrangement along the secretory pathway. *Plant Cell*.

[30] Verma DPS, Hong Z (2001). Plant callose synthase complexes. *Plant Molecular Biology*.

[31] Lerouxel O, Cavalier DM, Liepman AH, Keegstra K (2006). Biosynthesis of plant cell wall polysaccharides—a complex process. *Current Opinion in Plant Biology*.

[32] Zeng W, Keegstra K (2008). AtCSLD2 is an integral Golgi membrane protein with its N-terminus facing the cytosol. *Planta*.

[33] Davis J, Brandizzi F, Liepman AH, Keegstra K (2010). Arabidopsis mannan synthase CSLA9 and glucan synthase CSLC4 have opposite orientations in the Golgi membrane. *Plant Journal*.

[34] Pear JR, Kawagoe Y, Schreckengost WE, Delmer DP, Stalker DM (1996). Higher plants contain homologs of the bacterial celA genes encoding the catalytic subunit of cellulose synthase. *Proceedings of the National Academy of Sciences of the United States of America*.

[35] Liepman AH, Nairn CJ, Willats WGT, Sørensen I, Roberts AW, Keegstra K (2007). Functional genomic analysis supports conservation of function among cellulose synthase-like a gene family members and suggests diverse roles of mannans in plants. *Plant Physiology*.

[36] Northcote DH (1972). Chemistry of plant cell wall. *Annual Review of Plant Physiology*.

[37] Bacic A, Harris PJ, Stone BA, Preiss J (1988). Structure and function of plant cell walls. *Biochemistry of Plants*.

[38] Meier H, Reid JSG, Loewus FA, Tanner W (1982). Reserve polysaccharides other than starch in higher plants. *Encyclopedia of Plant Physiology*.

[39] Piro G, Lenucci M, Dalessandro G (2000). Ultrastructure, chemical composition and biosynthesis of the cell wall in Koliella antarctica (Klebsormidiales, Chlorophyta). *European Journal of Phycology*.

[40] Suzuki S, Li L, Sun Y-H, Chiang VL (2006). The cellulose synthase gene superfamily and biochemical functions of xylem-specific cellulose synthase-like genes in Populus trichocarpa. *Plant Physiology*.

[41] Ubeda-Tomas S, Edvardsson E, Eland C (2007). Genomic-assisted identification of genes involved in secondary growth in Arabidopsis utilising transcript profiling of poplar wood-forming tissues. *Physiologia Plantarum*.

[42] Goubet F, Barton CJ, Mortimer JC (2009). Cell wall glucomannan in Arabidopsis is synthesised by CSLA glycosyltransferases, and influences the progression of embryogenesis. *Plant Journal*.

[43] Obembe OO, Jacobsen E, Visser RGF, Vincken J-P (2006). Cellulose-hemicellulose networks as target for in planta modification of the properties of natural fibres. *Biotechnology and Molecular Biology Review*.

